# Diet composition impacts eQTL discovery across multiple tissues in baboons

**DOI:** 10.1016/j.xgen.2024.100524

**Published:** 2024-03-13

**Authors:** Rachel M. Petersen, Amanda J. Lea

**Affiliations:** 1 Department of Biological Sciences, Vanderbilt University, Nashville, TN, USA; 2 Vanderbilt Genetics Institute, Vanderbilt University, Nashville, TN, USA; 3 Vanderbilt Evolutionary Studies Initiative, Vanderbilt University, Nashville, TN, USA

## Abstract

Understanding how genetic variation impacts gene expression is a major goal of genomics; however, only a fraction of disease-associated loci have been demonstrated to impact gene expression when cells are in an unperturbed “steady state.” In this issue of *Cell Genomics*, Lin et al.^1^ investigate how exposure to a particular cellular context (i.e., a high-cholesterol, high-fat diet) can enhance our ability to identify new regulatory variants through longitudinal sampling of three tissue types in the baboon.

## Teaser

Understanding how genetic variation impacts gene expression is a major goal of genomics; however, only a fraction of disease-associated loci have been demonstrated to impact gene expression when cells are in an unperturbed “steady state.” In this issue of *Cell Genomics*, Lin et al. investigated how exposure to a particular cellular context (i.e., a high-cholesterol, high-fat diet) can enhance our ability to identify new regulatory variants through longitudinal sampling of three tissue types in the baboon.

## Main text

Understanding the path between non-coding genetic variation and organismal phenotype is a major goal of genomics. One popular approach for gaining traction on this problem is to map relationships between genetic variation and intermediate molecular phenotypes, such as gene expression (e.g., expression quantitative trait loci [eQTLs]). However, while eQTLs have now been mapped across a variety of datasets and cell types, they often fail to colocalize with non-coding genetic variants implicated in complex traits via other methods (e.g., genome-wide association studies [GWASs]).^2^^,^^3^ One potential reason for this difficulty is that certain eQTLs may only become active in particular cellular environments. For example, an allele may strongly affect expression when the cell is responding to an immune challenge or experiencing an active stress response, but otherwise has minimal effects on transcription. This suggests that eQTLs identified in easily accessible tissues (e.g., blood) when cells are in an unperturbed “steady state” may only account for a small fraction of the eQTLs present in the genome. While there is consequently widespread interest in understanding how genotype impacts gene expression under different cellular conditions, termed genotype-by-environment (GxE) interactions, it is often difficult to identify these interactions with high power. Study designs that are well suited to mapping eQTLs in a GxE interaction framework will offer important new insight into functional regulatory variation.

**Figure 1:**
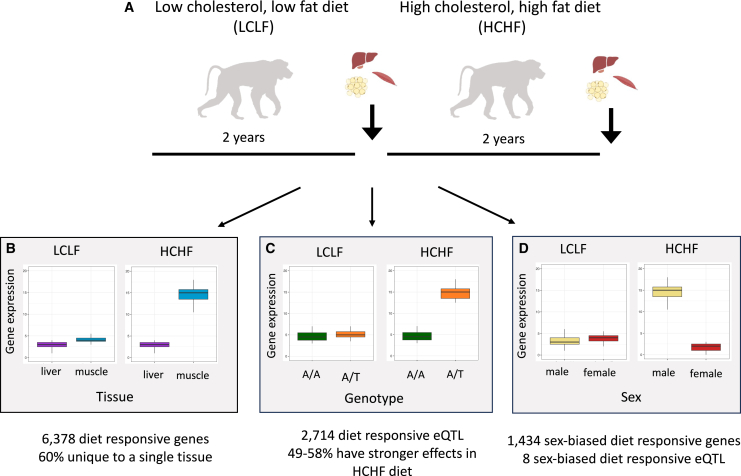
Study design and summary of main findings in Lin et al. (A) Longitudinal study design sampling multiple tissues before and after exposure to a high-cholesterol, high-fat diet. (B–D) Tissue heterogeneity in gene expression (B), context-dependent discovery of eQTLs (C), and sex-biased gene expression and eQTL discovery (D) following diet manipulation.

In this issue of *Cell Genomics*, Lin et al. used a controlled diet manipulation and within-subjects study design to map eQTLs in a GxE interaction framework across three tissues in captive baboons.^1^ The study consisted of 99 baboons, approximately half male and half female, who were fed a low-cholesterol, low-fat (LCLF) diet for 2 years, after which they biopsied adipose, muscle, and liver tissues from each individual. These same individuals were then fed a high-cholesterol, high-fat (HCHF) diet for 2 years and biopsied again, allowing for within-individual comparisons of gene expression before and after exposure to the HCHF diet (Figure 1A). Lin and colleagues found 6,378 diet responsive genes that differed in their expression levels between the LCLF and HCHF conditions, 60% of which were unique to a single tissue (Figure 1B). Across tissues, the study identified 12,402 eQTLs, of which, 2,714 differed in the presence, magnitude, or direction of effects between diet conditions (diet-responsive eQTLs, Figure 1C). Diet-responsive eQTLs were highly tissue specific, with only 6% shared between tissues in comparison to the 29%–38% of eQTLs shared between tissues in other studies of steady-state eQTLs.

Performing this study *in vivo* at the organismal level also allowed the authors to explore potential sex differences in the genetic basis of gene regulation, as sex differences in hormones, energy expenditure, social behavior, etc. may also contribute to the cellular environment, thereby influencing the identification of eQTLs. Adipose tissue showed the greatest sex-biased expression following diet manipulation, and although sex differences in the genetic regulation of gene expression were rare, the authors found eight sexually dimorphic diet-responsive eQTLs, including loci that had opposite effects in males versus females (Figure 1D). Finally, Lin and colleagues translated diet-responsive eQTLs to variants associated with metabolic disease in humans and observed qualitative differences in the genomic locations and connectivity of these context-specific eQTLs compared to steady-state eQTLs.

The longitudinal *in vivo* approach of this study makes it a powerful method to identify tissue heterogeneity in context-specific eQTLs, which has previously often been limited to cross-sectional or *in vitro* studies using tissue-specific cell lines. In addition to this strength, this study benefitted from the use of non-human primates as a model organism in other important ways. The power to detect eQTLs is associated with the proportion of each genotype present in the population. In baboons, alternate alleles are generally found at higher frequencies than they are in humans,^4^ creating greater power to detect genetic effects. In line with these predictions, the present study was able to identify 2,714 eGenes using 3 tissues from 99 baboons, which, according to published human datasets, would have required many more human samples for the same discovery power.^5^ The controlled environment that can be maintained in captive non-human primate studies also bolsters the ability to isolate stimuli and collect less-accessible cell types. Lastly, the genetic similarity between humans and non-human primates allows for interpretability with regard to human biological annotations, making them a powerful model to explain the molecular basis of metabolic disease in humans.

The thoughtful experimental design by Lin et al. offers an exciting jumping-off point for future eQTL discovery in non-human primates and also provides insight into the importance and properties of GxE interactions more generally. An important feature of studying context-specific eQTLs in whole organisms as opposed to in cell cultures is that complex pathways connecting stimuli and response can be observed. For example, a previous captive diet manipulation study of cynomolgus macaques found that a “Westernized diet” containing higher amounts of saturated fat was associated with higher levels of anxiety and less social integration,^6^ suggesting that links between diet and physiology may sometimes be mediated through changes in behavior. Other organism-level manipulations, such as experimental manipulations of social rank, are known to impact gene expression^7^ and could be fruitful for further within-subject studies of context-dependent eQTLs. Finally, Lin et al. add to an ongoing conversation about how context-specific eQTLs and steady-state eQTLs differ in both their genomic location and complexity, finding that the former may be more akin to GWAS hits and thus relevant to organism-level traits and disease.^2^ Thus, this paper presents one powerful approach to explain the regulatory relevance of GWAS hits that have thus far not been linked to steady-state eQTLs. In addition to expanding context-dependent eQTL studies to different tissue types and even more cellular contexts, other powerful genomic tools are emerging that can complement standard practices and give us greater insight into mechanisms such as multiplexed massively parallel reporter assays, CRISPR gene editing, and single-cell approaches. As the genomic toolbox continues to expand, thoughtful experiments utilizing multiple eQTL discovery and validation methods will be important for linking genetic variants to organism-level traits or disease.
